# Nano-Sized NiO Immobilized on Au/CNT for Benzyl Alcohol Oxidation: Influences of Hybrid Structure and Interface

**DOI:** 10.3390/molecules26206276

**Published:** 2021-10-16

**Authors:** Yixue Zhou, Fengxiang Shan, Sihan Yang, Jingjie Luo, Changhai Liang

**Affiliations:** Laboratory of Advanced Materials & Catalytic Engineering (AMCE), School of Chemical Engineering, Dalian University of Technology, Panjin 124221, China; Aquavit@mail.dlut.edu.cn (Y.Z.); sfx2020dlut@163.com (F.S.); yangsihan0513@163.com (S.Y.)

**Keywords:** Au-Ni catalysts, metal distribution, alcohol oxidation, colloid immobilization

## Abstract

Tiny gold nanoparticles were successfully anchored on carbon nanotubes (CNT) with NiO decoration by a two-step synthesis. Characterizations suggested that Ni species in an oxidative state preferred to be highly dispersed on CNT. During the synthesis, in situ reduction by NaBH_4_ and thermal treatment in oxidation atmosphere were consequently carried out, causing the formation of Au-Ni-O_x_ interfaces and bimetal hybrid structure depending on the Ni/Au atomic ratios. With an appropriate Ni/Au atomic ratio of 8:1, Ni atoms migrated into the sub-layers of Au particles and induced the lattice contraction of Au particles, whilst a higher Ni/Au atomic ratio led to the accumulation of NiO fractions surrounding Au particles. Both contributed to the well-defined Au-Ni-O_x_ interface and accelerated reaction rates. Nickel species acted as structure promoters with essential Au-Ni-O_x_ hybrid structure as well as the active oxygen supplier, accounting for the enhanced activity for benzyl alcohol oxidation. However, the over-layer of unsaturated gold sites easily occured under a high Ni/Au ratio, resulting in a lower reaction rate. With an Au/Ni atomic ratio of 8:1, the specific rate of AuNi_8_/CNT reached 185 μmol/g/s at only 50 °C in O_2_ at ordinary pressure.

## 1. Introduction

Efficient gold catalysts have been frequently designed and investigated for heterogeneous reactions since the first discovery of their excellent activity [1,2,3,4]. Great progress has been made for their industrial applications towards target products with long-term stability [5,6,7]. Up to now, a series of strategies have been proposed to enhance the selectivity of product and stability during thermal treatments and reactions. A routine method is the modulation of electronic and surface structure triggered by specific metal–support interfaces [8].

In recent years, much attention has been paid to Au-M bimetallic particles, concerning their unique and flexible structure that can be manually designed for heterogeneous reactions [9,10,11,12]. Bimetallic catalysts with contrivable structure were reported easily formed by gold chemically complexing with precious or transition metals such as Cu, Ni, Pd, Ru, Pt, etc. [13,14]. Transition metals are more often designed with alternative methods considering their high availability and decent price. On the other hand, the as-formed metal oxides facilitate the supplementing of active oxygen species during oxidations.

Different from other transition metals and precious metals that more easily form alloys with Au, Au-Ni-O_x_ complex is readily generated under thermal treatment and reaction conditions. Alloy could still be formed between Au and Ni-containing species in the presence of harsh reduction conditions. Nishikawa et al. [15] synthesized the Au-Ni catalyst by reducing the Au(OH)_3_-Ni(OH)_2_-NiCO_3_ co-precipitates in H_2_ atmosphere at 300 °C. Ni^0^ and Au-Ni alloy sites were suggested to co-exist as the active sites, and Au atoms highly dispersed with Ni domains were confirmed beneficial for the hydrogenolysis of alcohols. However, it was very hard to determine the real effect of Ni-containing species, due to the fact that most Au particles aggregated during the reduction and only a small amount of Au-Ni alloy was successfully obtained. It was also reported that the initial Ni catalyst was metastable and was easily covered by its own oxides in situ formed by exposed in-air or reaction conditions. The moderation of Ni by Au atoms was confirmed effectively to prevent the surface Ni^0^ species from being oxidized [16,17]. Kyriakou et al. [17] supported the Ni_50_Au_50_ alloy particles on α-Al_2_O_3_ for the CO + NO reaction. The active sites were contributed by metallic atoms, while oxidized NiO was proved catalytically inert. However, bimetal structure easily evolved, and substantial oxidation of Ni was evidenced to have occurred during reaction. The most incomprehensible part was that no direct link between the new formation of NiO and deactivation of the catalyst was observed, although the active sites was completely changed. On the contrary, Xu et al. [18] designed the Au-Ni nanocatalysts consisting of Au particles with NiO structure to form the “metal oxide-on-Au” inverse catalyst for CO oxidation. The specific structure not only enhances the catalytic performance of gold but also prevents the sintering of Au nanoparticles at elevated temperatures. Au nanoparticles decorated with highly dispersed NiO nanopatches were proved to be the active architecture with maximized boundaries of Au contacting NiO. According to the current investigations, controversies still exit. Au-Ni alloy structure was preferred and announced active for oxidation reactions by many researchers concerning the assumable uniform interfaces between Au and Ni atoms. However, the alloy structure evolved drastically during reaction. Ni species in oxidative state seems to be in a stable condition for oxidation reactions and cannot be ignored. Under such circumstance, it is wondered if the formation of an Au-Ni hybrid synergy/interface instead of the specific metastable bimetal/alloy structure is more essential for the reaction. The real functions of Ni species for reactions and the design of Au catalysts are still in need of investigation.

Herein in this work, a two-step colloidal technique was applied to synthesize a series of bimetallic AuNi_x_ catalysts using carbon nanotubes (CNT) as support. It has to be mentioned that CNT material has very good mechanical strength but easily controllable surface properties compared with other supporting materials. Different from most reducible metal oxides, pure CNT without specific functionalization generally displayed poor activity for many reactions, and the initial forces of AuNi_x_ catalysts could be mostly unraveled. There was no harsh reduction thermal treatment involved, and calcination was applied to remove the residual organic compounds and keep the high dispersion of oxidized NiO on surface. Their catalytic performances for the selective oxidization of benzyl alcohol to benzaldehyde were studied by modulating the atomic ratio of Au/Ni. Characterizations were carried out to understand the existing architecture of Ni species and the constructed Au-Ni hybrid interaction. Strong metal–support interaction between fine Au particles (2.6 nm) and support was facilitated in the presence of highly dispersed NiO. The distribution state and chemical structure of Ni and Au compositions can be deliberately modulated and correlated with the catalytic activity.

## 2. Results and Discussion

### 2.1. Selective Oxidation of Benzyl Alcohol

**Figure 1:**
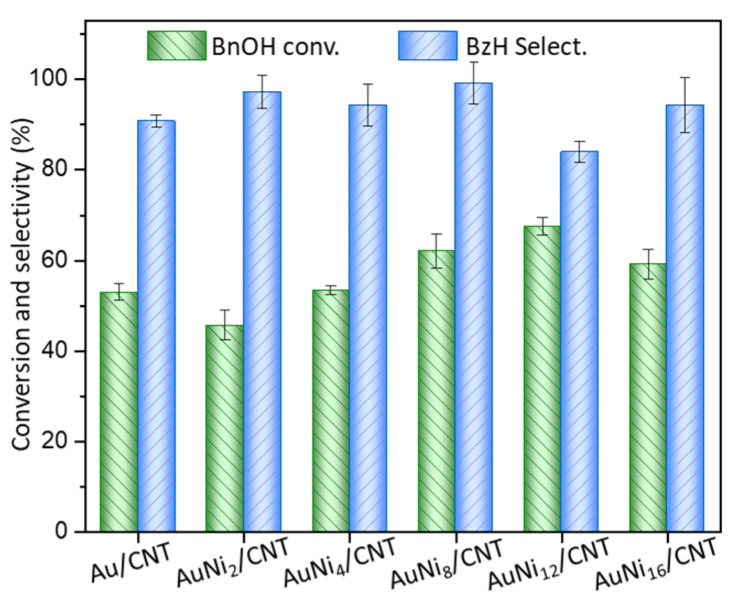
Catalytic performances of Au/CNT promoted by Ni-containing species. Reaction conditions: 50 °C for 3 h, O_2_ flow rate of 40 mL/min, 10 mg catalyst, alkali/BnOH = 0.42:1, BnOH/Au = 650:1 (molar ratio). BnOH—benzyl alcohol, BzH—benzaldehyde.

A series of AuNi_x_/CNT catalysts decorated by Ni species were prepared by a two-step synthesis. CNT decorated by Ni species was preliminarily synthesized by ultrasound-assisted impregnation, and Au particles were subsequently loaded by the colloid immobilization method. The series of AuNi_x_/CNT catalysts were used for the selective oxidation of benzyl alcohol to produce benzaldehyde (Figure 1). Under the current reaction conditions, benzyl alcohol can be transformed into benzaldehyde with a small amount of benzoic acid as by-product. The monometallic Au/CNT catalyst displays 52.9% conversion of benzyl alcohol with 90.0% selectivity of benzaldehyde. After doping a small amount of Ni species, the conversion of benzyl alcohol slightly decreases to 45.0% using AuNi_2_/CNT, with 97% selectivity of benzaldehyde. As the Ni/Au molar ratio further raises, conversion of benzyl alcohol continuously increases to 62.0% and 67.4% using AuNi_8_/CNT and AuNi_12_/CNT, with the corresponding benzaldehyde selectivity reaching 99.0% and 83.9% at only 50 °C. However, further increasing the Ni content in the AuNi_16_/CNT sample oppositely leads to decreasing conversion of benzyl alcohol to 58.5%.

**Figure 2:**
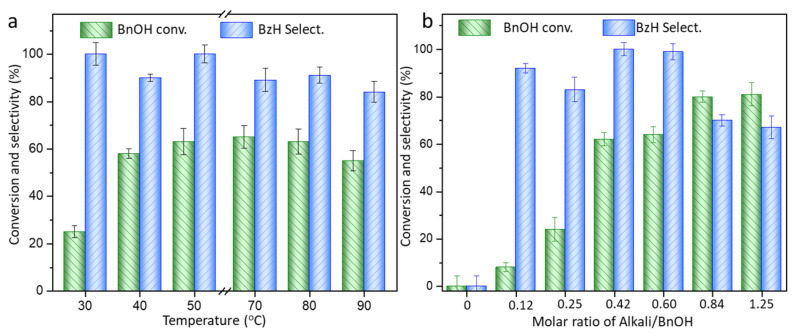
Catalytic performance of the AuNi_8_/CNT catalyst as a function of reaction temperature (**a**) and the alkali dosage (**b**). Reaction condition: Reaction at 50 °C for 3 h, O_2_ flow rate of 40 mL/min, 10 mg catalyst, alkali/BnOH = 0.42:1, BnOH/Au = 650:1 (molar ratio), if not specified. BnOH—benzyl alcohol, BzH—benzaldehyde.

The catalytic performance of the typical AuNi_8_/CNT as a function of temperature is displayed in Figure 2. The benzyl alcohol starts to be transferred at only 30 °C with 25.5% conversion of benzyl alcohol. It is sharply accelerated at 40 °C and arrives at a balance at about 50 °C, with 62% conversion and benzaldehyde selectivity of 99%. Benzaldehyde exhibits a catalytic selectivity higher than 80% for different samples at 50 °C. As the reaction temperature further raises to 90 °C, only 82% selectivity of benzaldehyde is detected. Higher temperature is beneficial for the activation and transformation of benzyl alcohol, due to endothermic nature of the oxidation process. However, excessive production of by-products is simultaneously generated due to the possible sped-up reaction rates.

The alkaline environment is intimately involved in the oxidation of benzyl alcohol to obtain benzaldehyde. The catalytic performances influenced by alkali dosage are reported in Figure 2b using AuNi_8_/CNT as typical catalyst. No observable conversion of benzyl alcohol can be detected in the absence of alkali, suggesting the indispensable base environment for selective oxidation under such mild conditions. The oxidation conversion of benzyl alcohol increases with the alkali dosage. Nevertheless, the selectivity of benzaldehyde volcanically raises as the molar ratio of alkali/BnOH changes from 0.12–1.25. The selectivity of benzaldehyde reaches around 99% with alkali/BnOH ratio of 0.42. Lolli et al. [19] suggested that benzyl alcohol underwent Cannizzaro reaction in the presence of high concentration of alkali base. The H on the -OH of benzyl alcohol bonded with water in the presence of alkali to form a tetrahedral intermediate (C_6_H_5_-CH_2_-O^−^) and facilitated the formation of benzaldehyde by subsequently losing the hydride ion [20]. However, the presence of excessive base environment could directly result in the deep oxidation of benzaldehyde into by-product benzoic acid. As the molar ratio of alkali/BnOH is higher than 0.84, the selectivity of benzaldehyde is reduced to 70%. Benzoic acid is accumulated as by-product under such conditions.

Compared with the monolmetalic Au/CNT and Ni/CNT, the AuNi_x_/CNT samples are preferable to transform benzyl alcohol, with higher selectivity towards benzaldehyde at only 50 °C. Due to this circumstance, the chemical composition and the initial structures of AuNi_x_/CNT samples with Ni decoration are subsequently studied.

### 2.2. Structure and Morphology of Catalysts

**Figure 3:**
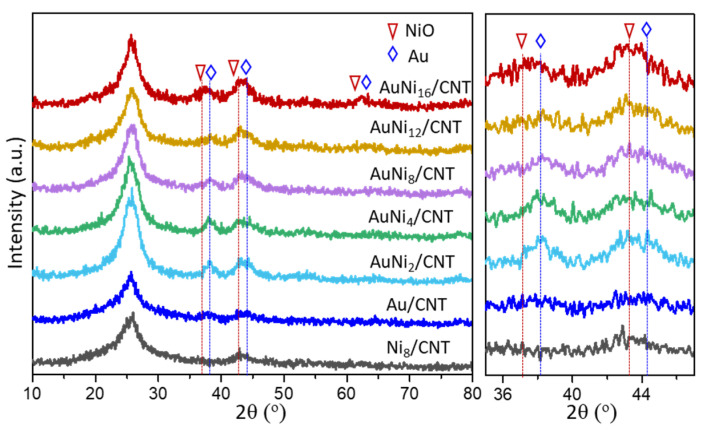
XRD patterns of different AuNi_x_/CNT samples.

The distribution and construction of NiO and Au crystals in the AuNi_x_/CNT samples directly manipulate chemical compositions and catalytic behaviors. Herein, the crystalline structures of AuNi_x_/CNT catalysts displayed by the XRD patterns are shown in Figure 3. The strong and broad diffraction peak at 25.9° is assigned to the (002) plane of amorphous carbon in CNT. The monometallic Ni_8_/CNT and Au/CNT references were also tested for comparison. Diffraction peak at 2θ = 43.3° can be observed and related to the (200) plane of bunsenite NiO (JCPDS No: 47-1049) [21]. Diffraction peaks at 38.2°, 44.4° and 64.6° can be assigned to the (111), (200), and (220) planes of face-centered cubic structure of Au (JCPDS No: 04-0784) [22].

In the series of AuNi_x_/CNT catalysts, characteristic peaks of NiO are not clearly observed at lower Ni/Au atomic ratio, probably due to their small sizes influenced by the neighboring diffraction peaks of Au crystals. The average crystal sizes are calculated according to Scherrer equation based on the peaks located at about 38.2°, which are 5.8 nm and 6.0 nm in Au/CNT and AuNi_2_/CNT. The average particle sizes of metal crystals gradually decrease with further increasing of the Ni/Au atomic ratio. The average crystal sizes in AuNi_8_/CNT and AuNi_12_/CNT are even minimized to 5.3 nm and 4.6 nm, suggesting the effectively suppressed sintering of particle sizes in the presence of Ni species. However, as the Ni/Au atomic ratio increases to 16:1 in AuNi_16_/CNT, diffraction peaks related to NiO appear at 37.2°, 43.3°, and 62.8°, corresponding to the (111), (200), and (220) crystal planes of NiO, respectively. The average size of metal particles reaches 7.8 nm. The much-sharpened diffraction peaks in AuNi_16_/CNT confirm the better growth of particles or crystallinity of NiO [23], which also evidences the impossible uniformity of Au-Ni-O_x_ hybrid structure with excessive Ni loading.

The XRD patterns in the range of 36–46° are subsequently enlarged, and surprisingly, a variation of 2θ in the range of 37.2–38.2° is observed with the increasing Ni/Au atomic ratio. Consolidation of specific Au-Ni-O_x_ hybrid structure may be generated instead of simple physical mixture of Au and NiO. Ni atoms have a chance to migrate into the surface lattice planes of Au to form the possible lattice constraint of Au crystals [24].

**Figure 4:**
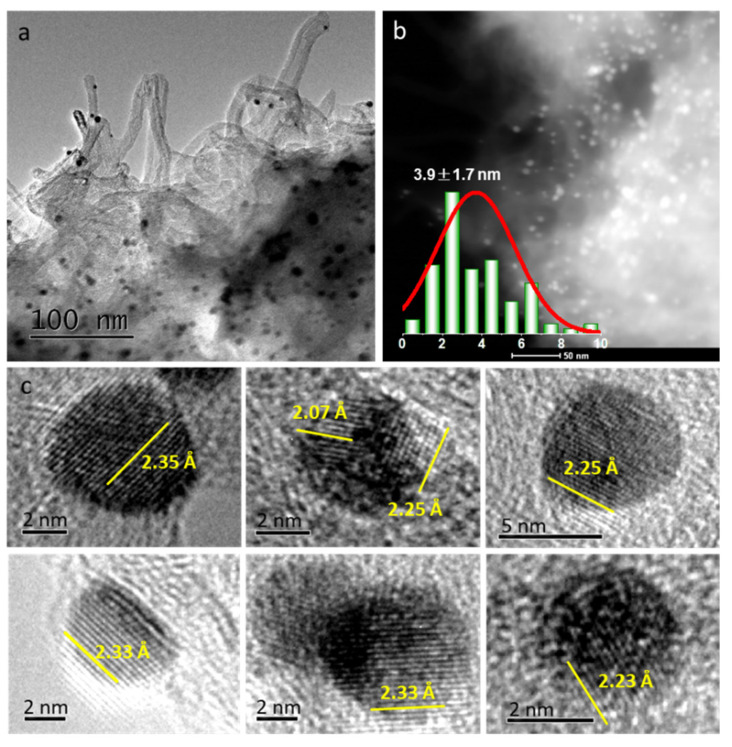
TEM image (**a**), HAADF-STEM images and size distribution (**b**), and high-resolution TEM images (**c**) of the typical AuNi_8_/CNT catalyst.

For the sake of metal morphology and surface distribution, typical TEM images of AuNi_8_/CNT are displayed in Figure 4. Metal nanoparticles in the AuNi_8_/CNT sample exhibit average size of 3.9 nm, which is lower than that of the monometallic Au/CNT (4.9 nm) (Figure S1 in supporting information) based on more than 200 particles. The presence of Ni species effectively prevents the sintering of nanoparticles during calcination. The HAADF-STEM elemental mappings of AuNi_8_/CNT (Figure S2) suggest randomly distributed Au particles on the surface of CNT. Either Ni or NiO crystal is absent in such samples, confirming the high dispersion of Ni species in the AuNi_8_/CNT sample.

High-resolution TEM images in Figure 4c display the typical morphology of particles dispersed on CNT surface. The presence of lattice plane with 2.35 Å confirms it as Au (111) face of Au nanoparticles [25]. However, it is surprising that interplanar distances in the range of 2.23–2.33 Å are observed on the edges of particles, which are between the lattice spaces of Au (111) (2.35 Å) and NiO (200) (2.07 Å) crystal planes [26]. In fact, it must be noted that the migration of single metal atoms was reported easily occurring during the synthesis, thermal treatment, and even reaction [27,28]. Combining the XRD results, it is inferred that Ni atoms may migrate into the surface atom layer of Au particles, causing the lattice constraint of the upper layer of Au particles [13]. These features are consistent with the classical variation in peak location in the XRD patterns.

**Figure 5:**
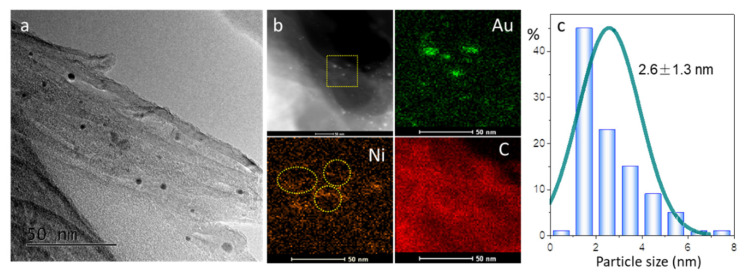
TEM image (**a**), HAADF-STEM elemental mappings (**b**), and the size distribution (**c**) of the AuNi_12_/CNT catalyst.

For the AuNi_12_/CNT catalyst at higher Ni/Au atomic ratio, the average particle size is further lowered to 2.6 nm (Figure 5). HAADF-STEM elemental mappings of AuNi_12_/CNT suggest the formation of small Au nanoparticles in the catalyst, with most of the Ni species still highly dispersed on the surface. However, a few Ni signals are found accumulating surrounding the Au particles randomly, as indicated by the dotted circles in Figure 5b. Luo et al. [29,30] suggested that excessive secondary metal species on inert carriers such as silica and carbon selectively deposited/migrated towards neighboring or mixing with Au particles during the synthesis and calcination processes. Herein, it is inferred that in contrast to the Ni atoms permeating into the sub-layer of Au particles in AuNi_8_/CNT, the excessive Ni content in AuNi_12_/CNT causes more Ni species to migrate onto the surface of Au particles [31].

### 2.3. Surface Chemistry and Properties

**Figure 6:**
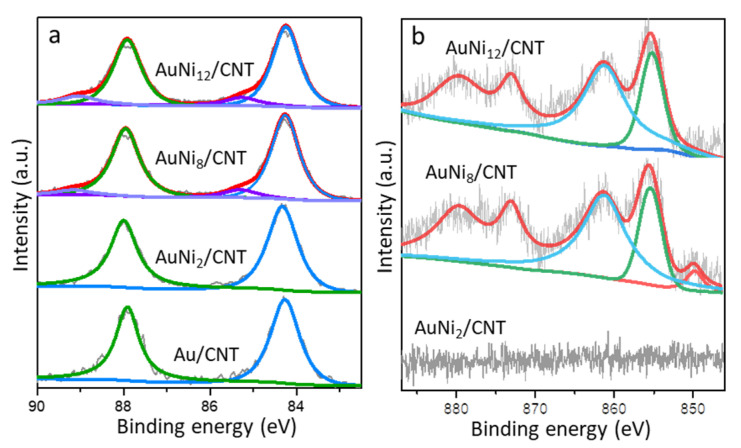
XPS spectra of the Au 4*f* (**a**) and Ni 2*p* (**b**) core level in typical catalysts.

The XPS spectra of Au 4*f* and Ni 2*p* core levels were performed to identify the surface chemical states of typical samples (Figure 6 and Table S1). The characteristic peaks maximized at 84.0 eV and 87.7 eV can be assigned to the Au 4*f*_5/2_ and 4*f*_7/2_ regions [32]. Only metallic Au^0^ exists on the surface of Au/CNT and AuNi_2_/CNT catalysts. Shoulder peaks appear after deconvolution at binding energy of 85.3 eV in both the AuNi_8_/CNT and AuNi_12_/CNT samples. They are assigned to the Au^δ+^ species (0 < *δ* < 1) [30], which was reported as a sign of strong Au–metal oxide (MO_x_) interaction [33]. It is evident that the co-existence of Ni species does not obviously change the chemical state of gold species at lower Ni/Au atomic ratio (≤2:1), since most of the Ni species randomly decorate the CNT surface. Combining the XPS results with the TEM images and XRD patterns, Ni species are not simply dispersing on or decorating the CNT as the Ni/Au ratio reaching 8:1. Contact between Au particle surface and Ni species is generated after the thermal treatment. Higher atomic ratio of Ni induces stronger interaction between Au and NiO due to the intimately constructed Au-Ni-O_x_ hybrid interface.

The XPS signal in the Ni 2*p* core level is not observed in AuNi_2_/CNT with low Ni/Au atomic ratio (Figure 6b). In the typical AuNi_8_/CNT sample, the overlapped characteristic peaks can be deconvoluted into two peaks at 850.1 and 855.3, corresponding to the presence of Ni^0^ and Ni^2+^, respectively [34]. The broad peak at 861.3 eV is ascribed to the satellite peak of Ni^2+^. However, only Ni^2+^ in oxidative state exists on the surface of AuNi_12_/CNT, and metallic Ni^0^ species is no longer detected. The surface information in Table S1 suggests the higher Au/C surface weight ratio of 2.9–3.3 wt% compared to the theoretical value (1.5 wt%) in different catalysts. It mainly results from the high dispersion of 2–6 nm Au particles on the outside surface of CNT, which could be mostly detected by the XPS technique applied to the CNT walls (diameter of 12 ± 4 nm). The surface atomic ratios between Ni and Au are 11.2 and 14.8 in the typical AuNi_8_/CNT and AuNi_12_/CNT catalysts. The slightly increasing Ni/Au ratio compared with the theoretical value (8:1 for AuNi_8_/CNT and 12:1 for AuNi_12_/CNT) reflects the higher surface distribution degree or smaller sizes of Ni than Au species. Concerning the XRD results and the synthesis method, it is inferred that Ni component is distributed on the surface of CNT mainly in the form of nickel oxide instead of forming uniform alloy with Au. However, metallic Ni^0^ is detected in the AuNi_8_/CNT, and the lattice constraint appears on the surface of Au nanoparticles as observed by XRD and high-resolution TEM images. The formation of small percentage of Ni^0^ in the AuNi_8_/CNT is consistent with the assumption that a few Ni atoms permeate into the sub-layer of Au particles. The even higher Ni/Au ratio in AuNi_12_/CNT results from the extrusion of Ni atoms to form NiO fractions surrounding Au particles, as revealed by the HAADF-STEM elemental mappings.

**Figure 7:**
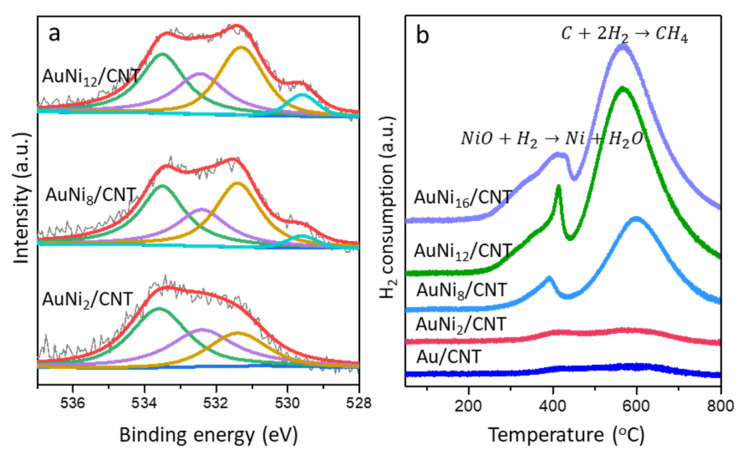
XPS spectra of the O 1*s* core level (**a**) and H_2_-TPR profiles (**b**) of different AuNi_x_/CNT samples.

The XPS spectra of O 1*s* region is shown in Figure 7a. The O 1*s* spectrum in the monometallic Au/CNT reference is deconvoluted into three peaks locating at 531.4, 532.4, and 533.8 eV, which are assigned to C=O, O-C=O, and C-O groups (Figure S3) [35], respectively. The O 1*s* signal in the AuNi_2_/CNT displays nearly identical distribution of oxygen species to Au/CNT. It is noteworthy that the AuNi_8_/CNT exhibits an additional peak located at 529.6 eV, ascribed to the lattice oxygen species supplied by oxidative nickel species [36]. The percentage of lattice oxygen species in AuNi_12_/CNT further raises due to the accumulation of nickel oxide. It has to be admitted that lattice oxygen plays an essential role during the oxidation reactions driven by gold catalysts [37], and the better conversion of catalytic activity by AuNi_x_/CNT can presumably be related.

As revealed by the XPS spectra, active lattice oxygen is greatly promoted by the introduction of Ni species. The percentage of lattice oxygen species varies as a function of the Ni atomic ratio, which would in turn result in the enhancement of redox properties as well as the catalytic behavior during the reaction. Herein, the H_2_-TPR profiles are illustrated in Figure 7b to present the redox behaviors of different AuNi_x_/CNT samples. For comparison, the data for Au/CNT are also given. No obvious reduction peak can be seen in the low-temperature region (<200 °C). It is reasonable that gold species are mainly existing as Au^0^. With the introduction of small amount of Ni species in AuNi_2_/CNT, broad and overlapped peaks in the range of 300–700 °C are observed and ascribed to the reduction of a few oxidized Ni species. As the Ni loading further increases, great reduction peaks are attributed to the AuNi_8_/CNT, AuNi_12_/CNT, and AuNi_16_/CNT samples. The first peak at 250–450 °C may result from small oxidized nickel species weakly bonding with CNT that are easily reduced by H_2_. The sharp peak at 500–700 °C may result from the reduction of NiO strongly interacting with CNT and even bulk NiO formed at a higher Ni ratio [38]. H_2_ consumption is calculated via different samples as 452, 3346, 3975, and 5623 μmol/g for AuNi_2_/CNT, AuNi_8_/CNT, AuNi_12_/CNT, and AuNi_16_/CNT, respectively. It also has to be mentioned that the increasing peak areas of AuNi_8_/CNT, AuNi_12_/CNT, and AuNi_16_/CNT are drastically higher than the theoretical consumption. This is mainly caused by the NiO promoted methanation of carbon in samples [39]. No matter how, the increasing H_2_ consumption by the first reduction region at lower temperature evidences the greatly enhanced supplement of O species in these samples.

It is now clear that two main aspects have been contributed by the Ni species during reaction. On the one hand, they act as common media for storage and supplementation of active oxygen species. The Ni/Au atomic ratio positively correlates with the oxygen storage/release ability induced by NiO. With the appropriate molar ratio of Ni/Au, an inseparable relationship is generated between the enhancement of catalytic activity and the existence of Ni species. Both XPS and TEM results indicate that Ni^2+^ species is highly dispersed on the surface of CNT. Specific connection between Au and Ni species can be generated easily, and active oxygen species are readily transferred from nickel oxide to the neighboring Au particles. Hence, the redox properties are largely improved by Ni decoration. On the other hand, Ni species act as structure promoters to trigger the modification of Au particle sizes and the Au-Ni-O_x_ hybrid interfaces. Appropriate Ni/Au ratio first induces the Ni atoms permeating into the sub-layer of Au particles and causes the lattice constraint of Au, and excessive Ni/Au leads to the formation of NiO fractions surrounding Au particles. There is not clear evidence of the formation of uniform Au-Ni alloy in all these samples. The collaboration of the Au-Ni-O_x_ hybrid structures accelerates the reaction rate using AuNi_x_/CNT with appropriate chemical ratios. For better comparison, the specific rate per gram of active gold per second was compared with the most reported results (Table S2). The specific rates are 185 and 208 μmol/g/s for AuNi_8_/CNT and AuNi_12_/CNT, which is among the efficient catalysts reported by literature [40,41,42]. No matter how, the balance exists between the exposure of Au sites and construction of Au-Ni-O_x_ hybrid structure. Even higher Ni/Au ratio results in the overlapping of unsaturated Au sites by the well-crystalized NiO. It also explains the sudden drop in conversion and the specific rate for AuNi_16_/CNT in Table S2.

## 3. Materials and Methods

### 3.1. Materials

Commercial CNT was supplied by Shandong Dazhan Nano Materials Co., Ltd. (length of 5–15 μm with the average diameter of 12 ± 4 nm, Binzhou, China). Before usage, CNT was purified with 37% HCl aqueous solution at room temperature for 24 h (1 g CNT to 40 mL HCl solution) to remove any residual metal species introduced during CNT manufacture.

Decoration of CNT by NiO: A series of Ni_x_/CNT catalysts with different Ni atomic ratios were prepared by the impregnation method, similar to the reported literature [43]. The concentration of the aqueous solution of nickel nitrite was fixed at 0.513 mol/L. Ultrasound treatment at room temperature was applied during the mixing of the Ni precursor and CNT for better metal dispersion. A total of 5 g purified CNT was dispersed in water with certain amount of nickel nitrate and maintained in an ultrasonic bath for 60 min. After being dried at 100 °C in an oven, the solid was ground and calcined in 25% O_2_/Ar at 300 °C for 3 h. The heating rate for calcination is 2 °C/min. After being cooled down to room temperature, the black powder was denoted as Ni_x_/CNT (x indicated the atomic ratio of Ni/Au; x = 2, 4, 8, 12, or 16).

Preparation of supported Au catalysts: Supported gold catalysts were synthesized by the colloidal mobilization process as reported by our previous work [44]. Colloidal gold particles were preliminarily synthesized and used as gold precursor. The aqueous solution of HAuCl_4_ (5.08 × 10^−2^ mol/L, 3mL) was mixed with 1% PVA ($\bar{M}=30,000–70,000$) aqueous solution (15 mL) and stirred at room temperature for 20 min. The fresh solution of NaBH_4_ (0.1 mol/L) was rapidly added into the above solution. The weight ratio of PVA/Au and molar ratio of NaBH_4_/Au were both kept at 5:1. A total of 2 g supporting material (purified CNT or Ni_x_/CNT) was poured into the colloid for 16 h under continuous stirring. The black powder was then filtered and washed with deionized water until free of chloride. After being dried overnight at 60 °C in an oven and calcined at 300 °C for 3 h under 25% (*v*/*v*) O_2_ balanced with Ar, the final catalysts were denoted as AuNi_x_/CNT (x indicated the atomic ratio of Ni/Au; x = 2, 4, 8, 12, or 16). The heating rate for calcination is 2 °C/min.

### 3.2. Characterizations

X-ray diffraction (XRD) analysis was measured on 7000S equipment (Simadzu, Berlin, Germany) with Cu Kα (λ = 1.5418 Å) source, enabling the measurement from 10° to 80° with the scanning rate of 3°/min. The average size was estimated using the Scherrer formula. X-ray photoelectron spectroscopy (XPS) measurements were obtained with the ESCALAB™ 250Xi (FEI, Hillsborough, OR, USA) using monochromatic Al Kα radiation. The binding energy was calibrated based on the C1*s* peak at 284.6 eV. Transmission electron microscopy (TEM) and high-angle annular dark-field (HAADF) images in the scanning transmission electron microscopy (STEM) mode were performed on the Tecnai G2 F30 (FEI, Hillsborough, OR, USA). Hydrogen temperature-programmed reduction (H_2_-TPR) measurements were accomplished using the Quantachrome Chembet 3000 Chemisorber (Quantachrome, Boynton Beach, VA, USA) to analyze the redox property of catalyst. Before each test, 50 mg sample was loaded and pretreated in Ar for 30 min to remove the contaminants and then cooled down to room temperature. The profiles were recorded from 50 °C to 800 °C (ramp rate of 10 °C/min) in 10% H_2_/Ar with the flow rate of 30 mL/min.

### 3.3. Evaluation of Catalytic Performance

The benzyl alcohol catalytic oxidation was conducted in a 25 mL three-necked flask equipped with an electronically controlled magnetic stirrer of oil bath connected to the continuous oxygen flow with 40 mL/min. The concentration of benzyl alcohol in this reaction was 1 wt%. The volume of reaction mixture in the three-necked flask was kept at about 10 mL. NaOH was used as the alkali with the molar ratio of alkali/BnOH as 0.42:1 if not specified. Before reaction, the reaction mixture of 10 mg catalyst, *p*-xylene as solvent, and benzyl alcohol were heated to the target value. After reaction, the liquid products were cooled down to room temperature, and the supernatant liquid after filtration was analyzed by a gas chromatograph (Agilent GC-7890, Agilent, CA, USA) equipped with a flame ionization detector (Agilent, CA, USA) and a 0.32 mm × 30 m HP-5 capillary chromatographic column (Agilent, CA, USA). Typical reaction products, the solvent (*p*-xylene), and the internal standard substance (biphenyl) can be clearly distinguished from each other by isolated chromatographic peaks (Figure S4). The ^13^C-NMR spectra of typical reaction mixture are displayed in Figure S5. The benzyl alcohol (BnOH) conversion and the selectivity of benzaldehyde (BzH) were calculated by the following equations using internal standard method:

(1) $$[Conv_{\mathrm{BnOH}}]=\frac{n_{0}-n_{\mathrm{BnOH}}}{n_{0}}\times 100\%$$

(2) $$\left[Sel_{\mathrm{BzH}}\right]=\frac{n_{\mathrm{BzH}}}{n_{0}-n_{\mathrm{BnOH}}}\times 100\%$$

where *n*_0_ is the initial mole number of benzyl alcohol (BnOH), and *n*_BnOH_ and *n*_BzH_ are the final mole numbers of BnOH and benzaldehyde (BzH), respectively.

## 4. Conclusions

In this work, a series of AuNi_x_/CNT catalysts were synthesized by two-step colloidal method for the selective oxidation of benzyl alcohol. Gold particles were anchored on the surface, and most of the fine NiO were dispersed on CNT instead of forming alloy with Au. Metallic gold species were dominant in the AuNi_x_/CNT catalysts independent of Ni decoration, while the distribution and the bimetal hybrid structure of particles were greatly modulated by the atomic ratios of Ni/Au. With a suitably low atomic ratio of Ni/Au, surface lattice contraction of Au was evidenced and induced by Ni atoms permeating into the sub-layer of Au particles. A higher amount of excessive Ni species caused the effusion of NiO fractions generated surrounding the Au particles. The Au-Ni-O_x_ hybrid structure could be constructed in both cases. However, the accumulation of NiO possibly covered the active gold sites and suppressed the reaction rate, breaking the balance between the exposed Au sites and the well-adjusted hybrid interface. With appropriate modulation, the decoration of Ni species easily facilitated the formation of Au-Ni-O_x_ hybrid structure, acting as both structure promoter and oxygen supplier for gold catalysts for the selective oxidation of benzyl alcohol. With NiO decoration, the oxidation of benzyl alcohol can be carried out under much lower temperature compared to the reported works. The specific rate of AuNi_8_/CNT reached 185 μmol/g/s at only 50 °C in O_2_ at ordinary pressure, catching up with the reported ones. The well-defined Au-Ni-O_x_ interface was evidenced in the samples with better catalytic performances.

## Data Availability

The raw data files are available from authors upon request.

## Supplementary Materials

The following are available online, Figure S1: TEM image and size distribution of Au/CNT catalyst, Figure S2: HAADF-STEM element mappings of the AuNi_8_/CNT catalyst, Figure S3: XPS spectra of the O 1s (c) core level of Au/CNT reference sample; Figure S4: A typical GC analysis graph by Au reflecting the chromatographic peaks of benzaldehyde, benzyl alcohol, and biphenyl (substance) in the reaction mixture; Figure S5: ^13^C NMR spectra of typical reaction mixture; Table S1: Surface composition of different gold catalysts; Table S2: Comparison of specific rate in the current work and the reported literature.

## Sample Availability

Samples of the compounds are available from the authors.
