# Supplementary material for: Nano-Sized NiO Immobilized on Au/CNT for Benzyl Alcohol Oxidation: Influences of Hybrid Structure and Interface
Source: Molecules. 2021 Oct 16;26(20):6276. doi: 10.3390/molecules26206276 (PMC8538636; doi:10.3390/molecules26206276)
Supplement: Supplementary file 1 [file molecules-26-06276-s001.zip › molecules-1391566-supplementary.pdf]

Supporting information

Nano-sized NiO immobilized on Au/CNT for benzyl alcohol oxidation: Influences of hybrid structure and interface

Yixue Zhou, Fengxiang Shan, Sihan Yang, Jingjie Luo,* Changhai Liang*

Laboratory of Advanced Materials & Catalytic Engineering (AMCE), School of Chemical Engineering, Dalian University of Technology, Panjin Campus, Panjin 124221, China

*Corresponding author. Tel/Fax: +86-411-84986353;

E-mail: changhai@dlut.edu.cn (C. Liang) & jingjie.luo@dlut.edu.cn (J. Luo)

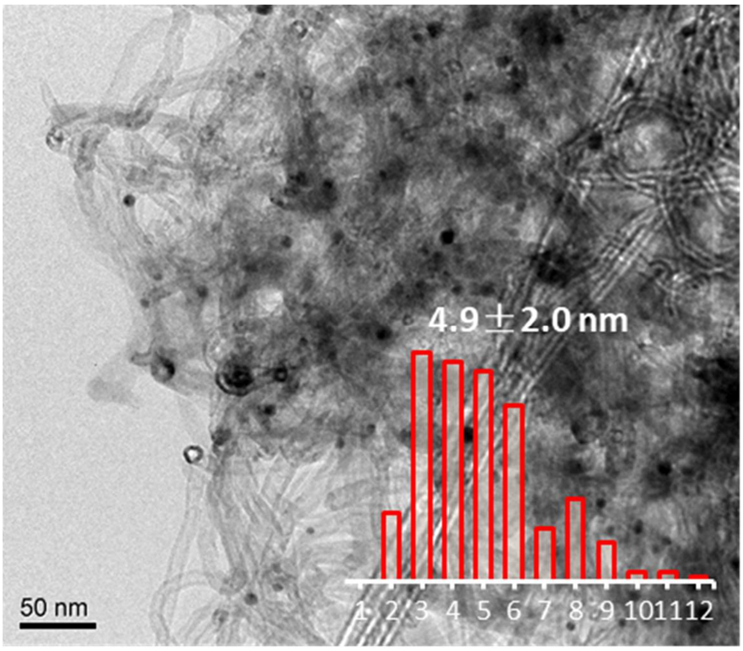

Figure S1. TEM image and size distribution of Au/CNT catalyst.

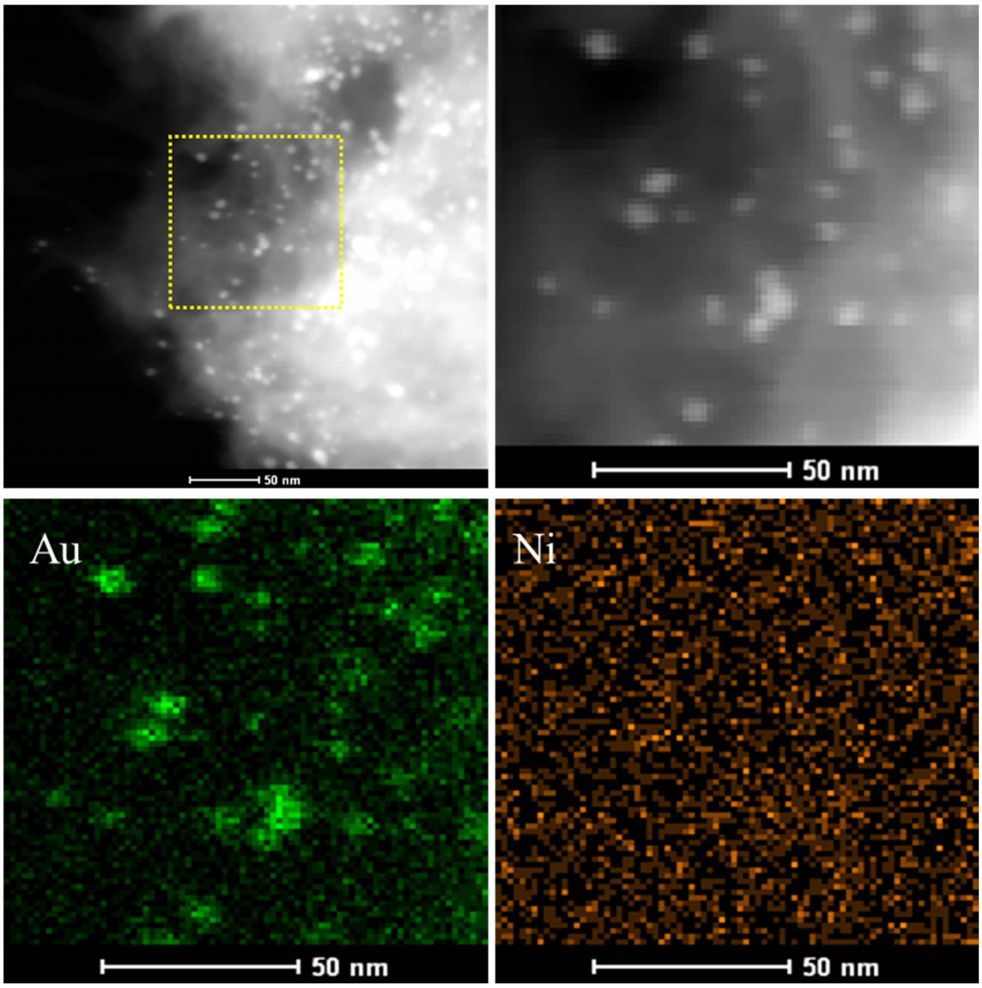

Figure S2. HAADF-STEM element mappings of the $AuNi_8$/CNT catalyst.

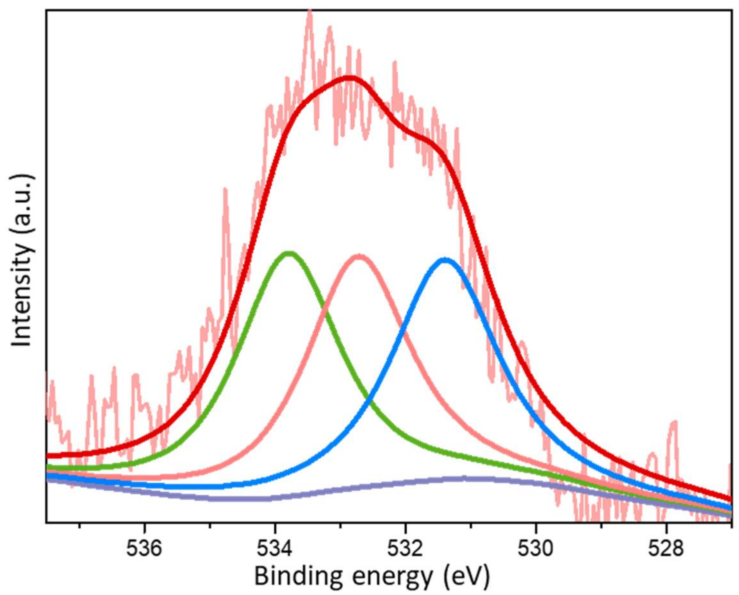

Figure S3. XPS spectra of the O 1s (c) core level of Au/CNT reference sample.

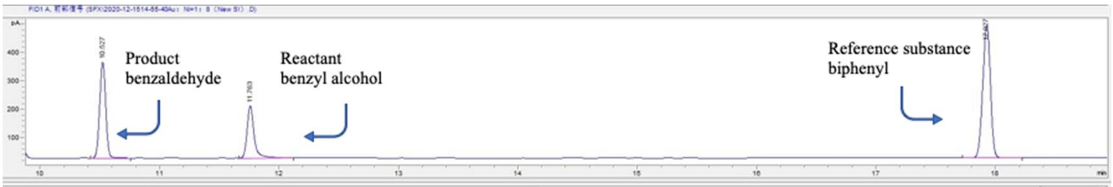

Product information：$AuNi_8/CNT$ (SI)
[1][2]Solvent：paraxylene
[3]Product：benzaldehyde
[4]Reactant：benzyl alcohol
[5]Reference substance：biphenyl

#	时间	峰面积	峰高	峰宽	对称因子	峰面积 %	类型
1	9.148	249055.3	23811.1	0.134	4.765	96.454	BB
2	9.418	5108.8	1654.9	0.047	1.063	1.979	BB
3	10.527	1197.3	339.5	0.0551	0.988	0.464	BB
4	11.763	751.9	183.6	0.0608	0.729	0.291	BB
5	17.927	2098.3	467.7	0.0713	1.095	0.813	BB

Figure S4. A typical GC analysis graph by Au reflecting the chromatographic peaks of benzaldehyde, benzyl alcohol, and biphenyl (substance) in the reaction mixture.

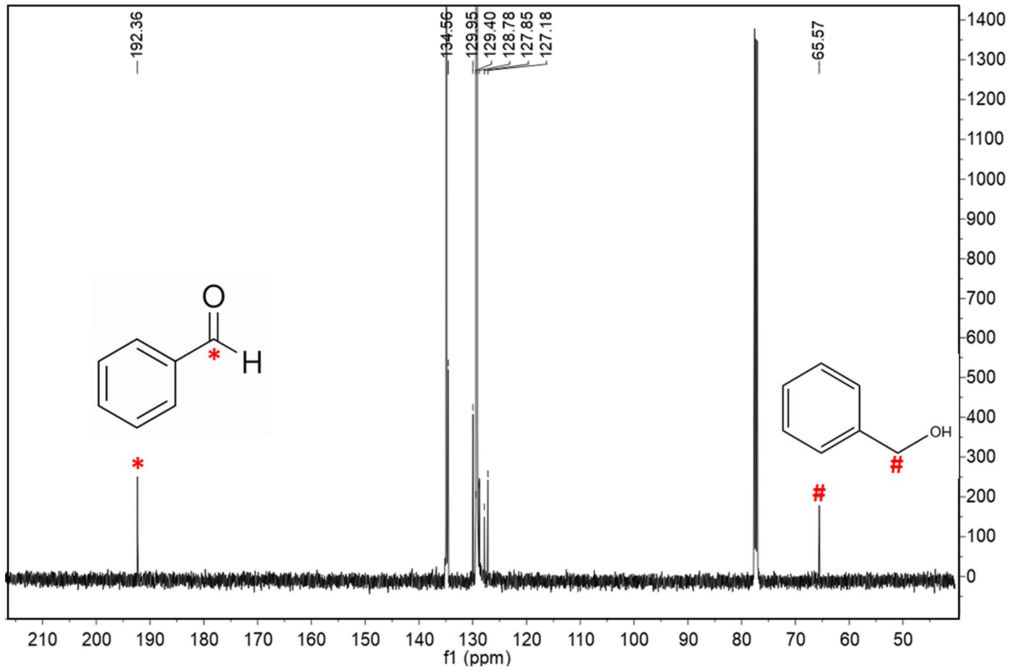

Figure S5. ^{13}C NMR spectra of typical reaction mixture.

Table S1 Surface composition of different gold catalysts.

Catalyst	Au 4*f*		Ni 2*p*		Au/C%[a]	Ni/Au[b]
	Au^0 (%)	$Au^{\delta+}$ (%)	Ni^0 (%)	Ni^{2+} (%)		
Au/CNT	100	0	n.a.	n.a.	3.1	n.a.
$AuNi_2$/CNT	100	0	n.a.	n.a.	2.9	n.a.
$AuNi_8$/CNT	90.1	9.9	17.4	82.6	3.4	11.2
$AuNi_{12}$/CNT	86.1	13.9	0	100	3.3	14.8

[a] Surface weight ratio of Au/C by XPS results.

[b] Surface atomic ratio of Ni/Au by XPS results.

Table S2 Comparison of specific rate in the current work and the reported literature

Catalyst	Reaction temperature	Specific rate (μmol/g/s)	Literature
Au/CNT	50 °C	163	This work
$AuNi_8/CNT$	50 °C	185	This work
$AuNi_{12}/CNT$	50 °C	208	This work
$AuNi_{16}/CNT$	50 °C	182	This work
0.5Au-CeZr	100 °C	370	[1]
RMC-Au	130 °C	212	[2]
Au/CZ	80 °C	106	[3]
Au−Pd/TiO_2	90 °C	1239	[4]
Au−Pd/TiO_2-001	120 °C	26002	[5]

References

1. Faqeeh, A. J.; Ali, T. T.; Basahel, S. N.; Narasimharao, K., Nanosized Samarium Modified Au-$Ce_{0.5}Zr_{0.5}O_2$ Catalysts for Oxidation of Benzyl Alcohol. *Mol. Catal.* **2018,** *456*, 10-21.

2. Oliveira, A. A. S.; Costa, D. S.; Teixeira, I. F.; Parreira, L. A.; Menini, L.; Gusevskaya, E. V.; Moura, F. C. C., Red Mud Based Gold Catalysts in the Oxidation of Benzyl Alcohol with Molecular Oxygen. *Catal. Today* **2017,** *289*, 89-95.

3. Olmos, C. M.; Chinchilla, L. E.; Villa, A.; Delgado, J. J.; Hungria, A. B.; Blanco, G.; Prati, L.; Calvino, J. J.; Chen, X. W., Size, Nanostructure, and Composition Dependence of Bimetallic Au-Pd Supported on Ceria-zirconia Mixed oxide Catalysts for Selective Oxidation of Benzyl Alcohol. *J. Catal.* **2019,** *375*, 44-55.

4. Hong, Y. L.; Jing, X. L.; Huang, J. L.; Sun, D. H.; Odoom-Wubah, T.; Yang, F.; Du, M. M.; Li, Q. B., Biosynthesized Bimetallic Au-Pd Nanoparticles Supported on TiO_2 for Solvent-Free Oxidation of Benzyl Alcohol. *ACS Sustainable Chem. Eng.* **2014,** *2* (7), 1752-1759.

5. Wang, Z.; Feng, J. J.; Li, X. L.; Oh, R. N.; Shi, D. D.; Akdim, O.; Xia, M.; Zhao, L.; Huang, X. Y.; Zhang, G. J., Au-Pd Nanoparticles Immobilized on TiO_2 Nanosheet as an Active and Durable Catalyst for Solvent-free Selective Oxidation of Benzyl Alcohol. *J. Colloid Interface Sci.* **2021,** *588*, 787-794.
